# Congenital anomalies of the kidney and urinary tract

**DOI:** 10.3389/fmed.2024.1384676

**Published:** 2024-07-15

**Authors:** Anfal Hussain Mahmoud, Iman M. Talaat, Abdelaziz Tlili, Rifat Hamoudi

**Affiliations:** ^1^Research Institute for Medical and Health Sciences, College of Medicine, University of Sharjah, Sharjah, United Arab Emirates; ^2^Clinical Sciences Department, College of Medicine, University of Sharjah, Sharjah, United Arab Emirates; ^3^Pathology Department, Faculty of Medicine, Alexandria University, Alexandria, Egypt; ^4^Department of Applied Biology, College of Sciences, University of Sharjah, Sharjah, United Arab Emirates; ^5^BIMAI-Lab, Biomedically Informed Artificial Intelligence Laboratory, University of Sharjah, Sharjah, United Arab Emirates; ^6^Division of Surgery and Interventional Science, University College London, London, United Kingdom

**Keywords:** CAKUT, kidney anomalies, metanephros, ureteric bud, ultrasonography

## Abstract

Congenital Anomalies of the Kidney and Urinary Tract (CAKUT) refer to a range of conditions that affect the kidney and urinary tract. These anomalies can be severe, such as kidney agenesis, or milder, such as vesicoureteral reflux. CAKUT affects over 1% of live births and accounts for 40–50% of cases of chronic kidney failure in children. The pathogenesis of CAKUT is caused by various environmental, genetic, and epigenetic factors that disrupt normal nephrogenesis. Environmental factors that can lead to CAKUT include maternal diabetes, obesity, malnutrition, alcohol consumption, or medications affecting kidneys development. Genetic factors can cause an imbalance in the metanephros and the ureteric bud interaction. Defects in specific genes such as PAX2, TBX18, NRIP1, REX, SIX2, BMP4, and chromosome 17 cause CAKUT. Over 50 genes have been identified as the root cause of this condition, with monogenetic variants causing up to 20% of all cases. CAKUTs can be diagnosed through fetal ultrasonography, but some anomalies may remain undetected. GWASs, Next Generation Sequencing for targeted and whole exome DNA sequencing may provide additional diagnostic methods. This review article highlights some the leading factors that cause CAKUT, which adversely affects kidney development and urinary tract function.

## Introduction

CAKUTs refer to congenital anomalies that affect the kidney and urinary tract structures, including kidneys, ureters, bladder, and urethra (1). The urinary tract development may be disturbed at any point in embryogenesis, causing developmental abnormalities with diverse manifestations (2).

CAKUT can be isolated (non-syndromic) or with other phenotypes that affect other organs (syndromic). Moreover, it can be observed as a bilateral malformation affecting both kidneys and a unilateral malformation that affects only one kidney. The anatomical classification of CAKUT is complex due to the variability in phenotype, including incomplete penetrance and variable expression (2). However, the advent of new genomic technology, which can detect sequence variations, has enabled scientists to categorize defects according to their genetic architecture (3).

Many genes are involved in embryonic kidney development, which is also mutant in CAKUT patients (1). Furthermore, Van der Ven et al. identified 40 monogenic mutations leading to CAKUT and hypothesized that there is a high frequency of CAKUT phenotypes caused by single gene variations (2).

These studies indicate the genetic heterogeneity impact on CAKUTs, ultimately discovering new fundamental pathways. Further studies are required to confirm the mutagenesis of several other genes associated with essential developmental pathways (4). Moreover, recent studies have demonstrated that environmental and epigenetic factors influence CAKUT occurrence (5). Hence, understanding the etiology underlining CAKUT will improve diagnostic strategies and discover advanced therapeutic approaches.

This review article aims to investigate the etiology of CAKUT by highlighting the genetic, epigenetic, and environmental factors that contribute to the development of the disease. Furthermore, exploring the development complexity of kidney and urinary tract and how this process perturbs.

## Epidemiology of CAKUT

CAKUT occurs in over 1% of live births, accounting for 23% of all birth defects (6), and implicating in 34–59 percent cases of chronic kidney disease. Additionally, CAKUT is a significant cause of kidneys failure since it occurs in 40–50% of pediatric patients and 7% of adults who suffer from chronic kidney failure (7, 8). CAKUT have been associated with an increased risk of urinary tract cancer in later life. Pathways affected by CAKUT have also been identified as potential contributors to the development of cancer (9).

## Development of CAKUT

**Figure 1:**
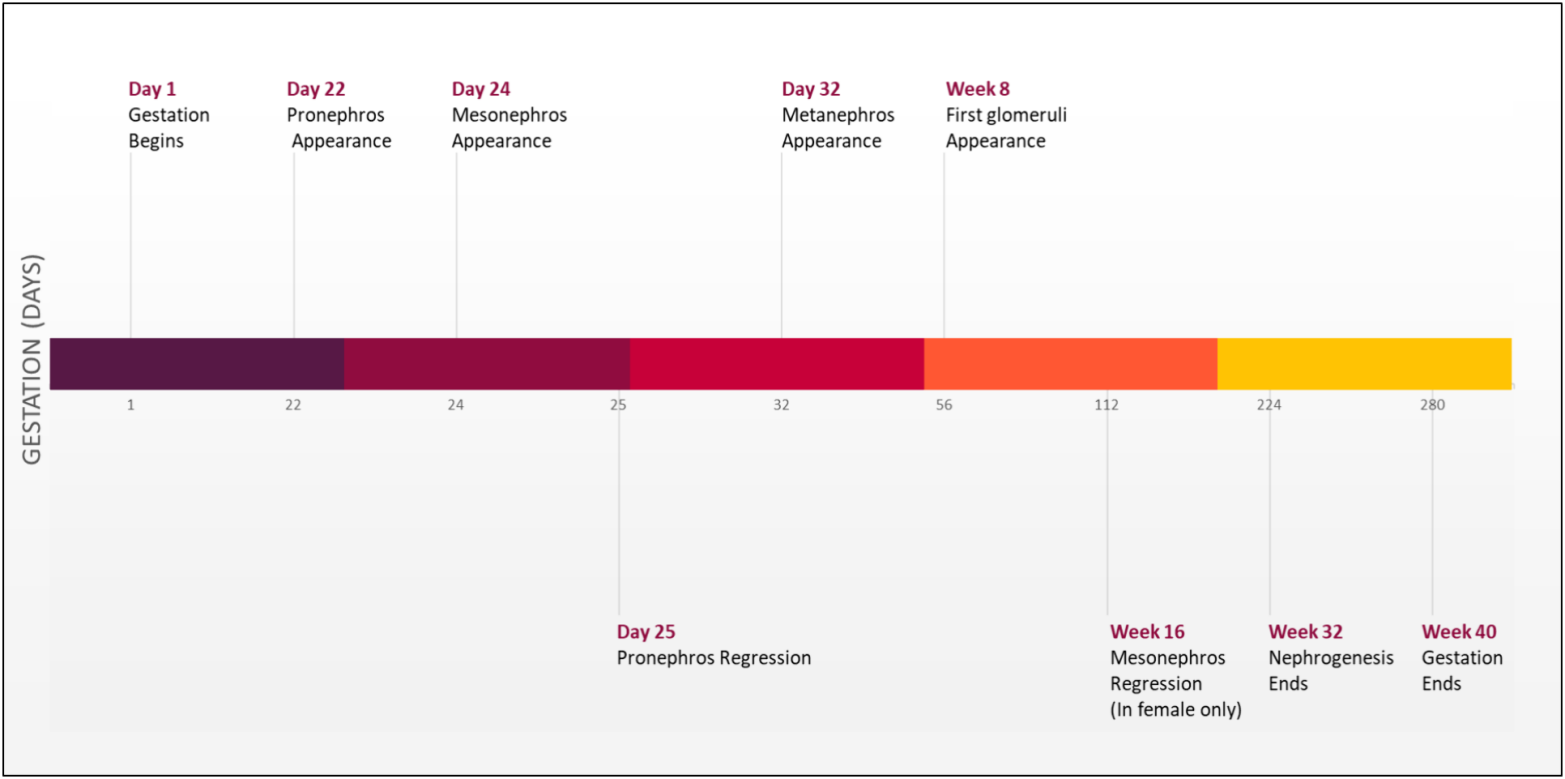
Timeline of kidney development in humans (5, 10).

Development of the urinary tract begins during the third week of gestation with the emergence of the nephrogenic cord from the intermediate mesoderm found on both sides of the embryo. This nephrogenic cord will form three structures that emerge sequentially on the dorsal body wall: the pronephros, mesonephros, and metanephros (Figure 1) (10).

Initially, pronephros will arise as a nonfunctional and immature kidney. Subsequently, this structure will regress, and the mesonephros will emerge to form a mesonephric duct that will elongate caudally to join the (cloaca), forming the urinary bladder. Additionally, a portion of the duct will protrude to form an epithelial tube known as a uretic bud (UB) surrounded by metanephric mesenchymal (MM). The UB will send a continuous reciprocal signal to the MM, initiating and enhancing the branching process of the UB and consequently forming an ultimate adult kidney by the end of week 32 of gestation (5, 11). A molecular signal disturbance between these two compartments may cause CAKUT manifestations (3). Severe cases of CAKUT can result in life-threatening conditions such as hydronephrosis, which leads to fibrosis and kidneys failure (1).

## Pathogenesis of CAKUT

Besides the molecular disturbance during embryonic development, CAKUT can also result from environmental, epigenetic, or genetic factors (12).

### Environmental factors

Several maternal conditions can affect the *in-utero* environment and lead to the development of CAKUT (12). These conditions include diabetes, chronic kidney disease, cancer, and obesity (13–15). Excessive intake of folic acid or deficiency in vitamin A during pregnancy is also associated with CAKUT manifestations (1, 3). Furthermore, some medications, such as angiotensin-converting enzyme inhibitors (ACE), can impair kidneys development (8, 16) Maternal malnutrition and a low-protein diet during pregnancy can have a detrimental impact on the developing kidneys system (5).

### Epigenetic factors

CAKUT epigenetic factors include DNA methylation, histone modifications, and non-coding RNAs. These epigenetic mechanisms control gene expression via activating or silencing regulatory genes essential for kidney and urinary tract development (3). Alterations to epigenetic marks that regulate developmental genes in the kidney and urinary tract can increase the risk of CAKUT phenotypes (12).

### Genetic factors

Multiple genetic factors may contribute to CAKUT, including monogenic mutations, copy number, and structural variations, leading to an imbalance in some proteins and dysregulation of essential receptors and signaling pathways. Furthermore, Van der Ven et al. hypothesize that there is a high frequency of CAKUT phenotypes caused by monogenic variations in genes related to kidney development (2). These monogenic mutations account for up to 20% of all CAKUT cases in human populations and include more than 50 genes causing CAKUT, such as PAX2, TBX18, NRIP1, REX, SIX2, and BMP4 (4).

## The current state of CAKUT genetic etiology

CAKUT occurs due to genetic factors, including defects in specific genes, rare genetic syndromes, and mutations in genes causing protein imbalance that affect kidney development or result in gene dysfunction. The gene variants linked to CAKUT include monogenic variants and copy number variations resulting in the down-regulation receptors, affecting various signaling pathways (12).

### Monogenetic variants

This section explores how variations in single genes increase the risk of CAKUT. Monogenic causes of CAKUT can be detected through various genetic screening techniques, such as single-gene screening, gene panels, and whole-exome sequencing. These techniques offer an in-depth analysis of specific genes or the entire exome, allowing for the identification of single-gene mutations responsible for CAKUT (12). TBX18 and NRIP1 genes are the common monogenic causes of CAKUT. They increase ureteric mesenchymal cell development defects and retinoic acid signaling, contributing to disease pathogenesis (4). PAX2 variants are a known cause of monogenic CAKUT. These mutations are associated with (syndromic CAKUT), which is associated with ocular anomalies. Monogenic variations are caused by a combination of familial genetic, epigenetic, and environmental factors. They account for up to 20% of all CAKUT cases in human populations and include around 68 monogenetic causes (39 dominant, 26 recessive, and 4 X-linked) (Table 1). Mutations in monogenic variants increase the prevalence of CAKUT genes in humans because they create genetic imbalances, alter genetic composition, and modify or delete genes (4).

**Table 1:** Genetic risks related to CAKUT, including autosomal dominant, recessive, and X-linked mutations.

	Gene	Protein	AD, AR, XL
1	ACE	Angiotensin I–converting enzyme	AR
2	AGT	Angiotensinogen	AR
3	AGTR1	Angiotensin II receptor	AR
4	AIFM3	Apoptosis-inducing factor, mitochondria associated 3	XL
5	BMP4	Bone morphogenic protein 4	AD
6	BNC2	Zinc finger protein basonuclin-2	AD
7	CBWD1	Cobalamin Synthetase W Domain–Containing Protein 1	AR
8	CHD1L	Chromodomain helicase DNA binding protein 1 like	AD
9	CHRM3	Muscarinic acetylcholine receptor M3	AR
10	CHRNA3	Cholinergic Receptor Nicotinic Alpha 3 Subunit	AR
11	COL4A1	Collagen alpha 1(IV) chain	AD
12	CRELD2	Cysteine Rich With EGF Like Domains 2	AD
13	CRKL	CRK-like proto-oncogene, an adaptor protein	AD
14	DAB1	DAB adaptor protein 1	AR
15	DSTYK	Dual serine/threonine and tyrosine protein kinase	AD
16	EYA1	Eyes absent homolog 1	AD
17	FGF20	Fibroblast growth factor 20	AR
18	FOXA2	Forkhead box A2	AR
19	FOXA3	Forkhead box A3: Hepatocyte nuclear factor 3-gamma: HNF3G	AD
20	FOXC1	Forkhead box protein C1	AD
21	FOXL2	Forkhead transcription factor	AD
22	FOXP1	Forkhead box protein P1	AD
23	FRAS1	ECM protein FRAS1	AR
24	FREM1	FRAS1-related ECM protein	AR
25	FREM2	FRAS1-related ECM protein	AR
26	GATA3	GATA binding protein 3	AD
27	GEN1	Holiday junction 5-prime flap endonuclease	AR
29	GFRA1	GDNF family receptor alpha 1	AR
30	GDF6	Growth/ Differentiation factor 6	AR
31	GREB1L	GREB1-like protein	AD
32	GRIP1	Glutamate receptor-interacting protein	AR
33	HNF1B	HNF homeobox B	AD
34	HOXA11	Homeobox protein Hox-A11	AR
35	HPSE2	Heparanase 2 (inactive)	AR
36	ITGA10	Integrin Alpha-10	AR
37	ITGA8	Integrin Alpha-8	AR
38	KAL1/ANOS1	Anosmin 1	XL
39	KIF4A	Kinesin family member 4 A	XLR
40	LRIG2	Leucine-rich repeats and Ig-like domains 2	AR
41	MUC1	Mucin 1	AD
42	NRIP1	Nuclear receptor-interacting protein 1	AD
43	PAX2	Paired box 2	AD
44	PBX1	PBX homeobox 1	AD
45	PLS3	PALSTIN 3: maintenance of normal kidney function	XLD
46	REN	Renin	AR, AD
48	RET	Proto-oncogene tyrosine-protein kinase receptor Ret	AD
49	ROBO2	Roundabout, axon guidance receptor, homolog 2 (Drosophila)	AD
50	SALL1	Sal-like protein 1 (also known as spalt-like transcription factor 1)	AD
51	SIX2	SIX homeobox 2	AD
52	SIX5	SIX homeobox 5	AD
53	SLC20A1	Solute carrier family 20 (phosphate transporter) member 1	AD
54	SLIT2	Slit homolog 2	AD
55	SON	Protein SON	AD
56	SOX17	Transcription factor SIX-17	AD
57	SRGAP1	SLIT-ROBO Rho GTPase activating protein 1	AD
58	TBC1D1	TBC1 domain family member 1	AD
59	TBX18	T-box transcription factor TBX18	AD
60	TBX6	T-box transcription factor TBX6	AD
61	TNXB	Tenascin XB	AD
62	TRAP1	Heat shock protein 75 (TNF receptor–associated protein 1)	AR
63	UMOD	Uromodulin	AD
64	UPK3A	Uroplakin	AD
65	VWA2	Von Willebrand factor A domain-containing protein 2	AR
66	WNT4	Protein Wnt-4	AD
67	WNT9B	Wingless—type MMTV integration site family, member 9B	AR
68	ZMYM2	Zinc finger MYM-type protein 2	AD

AD, autosomal dominate; AR, autosomal recessive; XL, linked to chromosome X; XLR, a recessive mutation linked to X chromosome; XLD, a dominate mutation linked to X chromosome.

### Copy number variations

Copy number variants (CNVs) are a significant genetic risk factor for CAKUT. CNVs can result in kidney and urinary tract defects by altering the expression of genes that are vital for proper kidney formation (3). Specifically, CNVs can lead to deletions or duplications of chromosomal regions that harbor genes necessary for kidney development. These alterations can cause an imbalance in gene expression, ultimately resulting in CAKUT phenotypes (1). Specific CNVs that have been associated with CAKUT include HNFB1/Hepatocyte nuclear factor 1-β (on Chromosome 17) and PAX genes (2). Although CNVs only account for about 5% of CAKUT cases, they provide evidence that other genetic variations can also lead to CAKUT disorders (1).

In CAKUT, severe phenotypes observed in offspring of healthy parents can be attributed to *de novo* dominant mutations in essential developmental genes or recessive mutations in genes that can tolerate reduced gene dosage. In contrast, familial cases of CAKUT, which represent 10–20% of cases, often exhibit incomplete penetrance as an autosomal dominant trait. This incomplete penetrance may arise from genetic or environmental modifiers that influence disease manifestation, hypomorphic mutations that partially impair kidney development, or disruption of genes and pathways essential for urinary tract development later in life (4). Structural disorders such as vesicoureteral reflux (VUR) and duplex collecting systems (DCS) are more prevalent because they have a lesser impact on overall survival. This is because they can either remain asymptomatic or spontaneously resolve over time. These common disorders may have a polygenic basis, meaning that they are caused by the combined effects of multiple common genetic variants. These variants have modest effects on an individual’s risk of developing the disorder, and they are less likely to be influenced by selective pressures (4).

## The phenotypic spectrum of CAKUT

Embryonic development of the urinary tract system is a complex process. CAKUT phenotypes may arise from disrupting this process (4). In particular, a disturbance in the communication between the metanephric mesenchyme (MM) and the ureteric bud (UB) that arises from the nephric duct can result in CAKUT (8). The location of ureteric bud (UB) emergence along the nephric duct is a crucial determinant of CAKUT phenotype. In the case of a low insertion of the UB, (vesicoureteral reflux) will result, while a high insertion results in (obstructive uropathy) (3). CAKUT phenotypes are highly variable between individuals, and multiple anomalies can co-occur within a single patient. CAKUT can occur alone (non-syndromic CAKUT) or in association with other syndromes and diseases (syndromic CAKUT). The severity of CAKUT varies widely, and the condition can cause a variety of structural abnormalities in the kidneys, ureters, and lower urinary tract (3).

### Anomalies of the kidneys

An early embryonic maldevelopment can result in several kidney parenchymal defects, including:

1. **Kidney agenesis**

**Figure 2:**
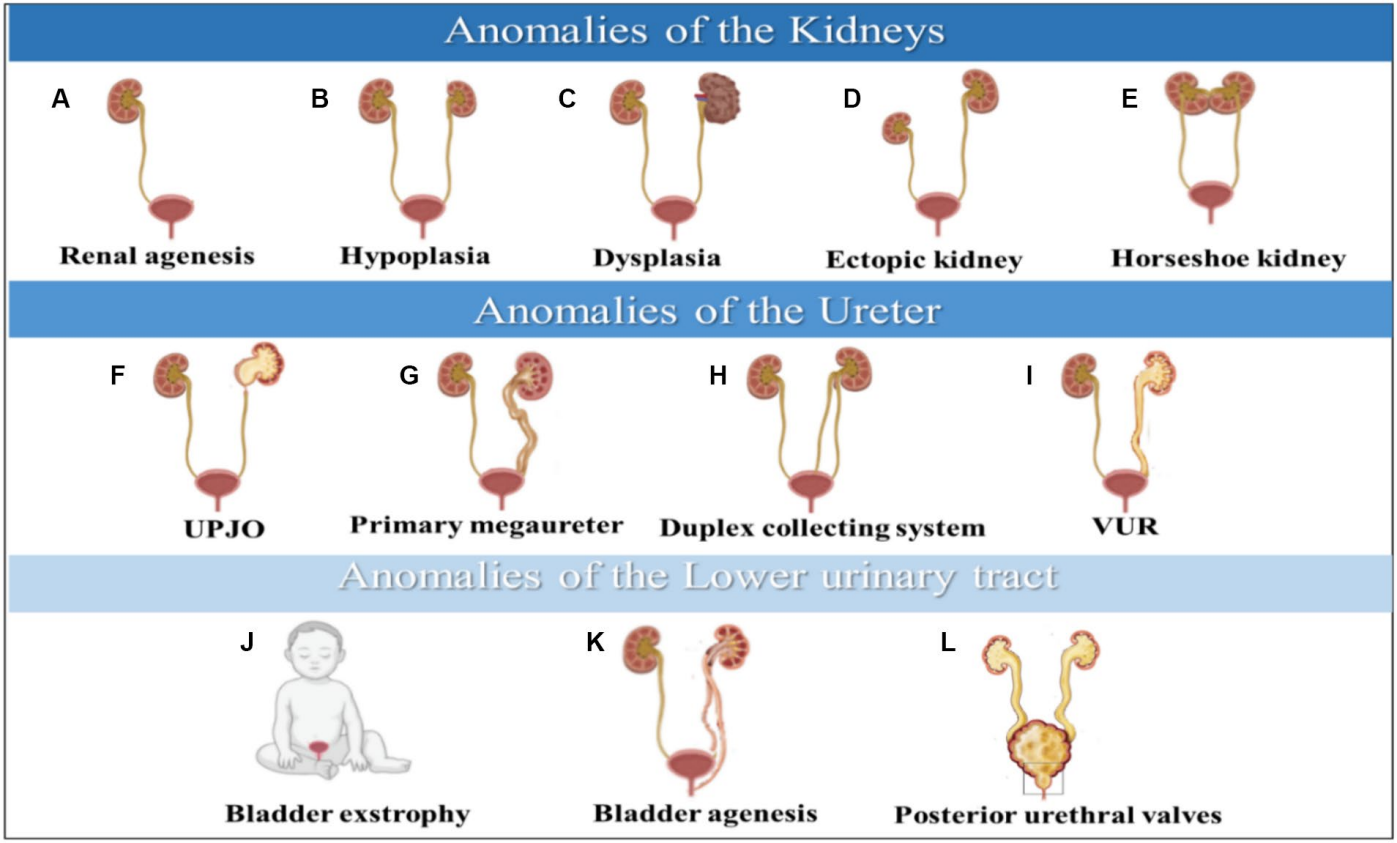
Anomalies of the kidneys are illustrated in **(A–E)**, anomalies of the ureter are shown in **(F–I)**, and anomalies of the lower urinary tract are depicted in **(J–L)**. CAKUT phenotypic spectrum includes kidney anomalies, ureter, and lower Urinary tract. **(A)** Renal agenesis, **(B)** Hypoplasia, **(C)** Dysplasia, **(D)** Ectopic kidney, **(E)** Horseshoe kidney, **(F)** UPJO; ureteropelvic junction obstruction, **(G)** Primary megaureter, **(H)** Duplex collecting system, **(I)** VUR; vesicoureteral reflux, **(J)** Bladder exstrophy, **(K)** Bladder agenesis, **(L)** Posterior urethral valves.

Kidney agenesis refers to a congenital anomaly characterized by the absence of one or both kidneys (Figure 2A). It can be isolated or part of multi-organ syndromes (5). The exact mechanisms underlying it are not fully understood, but genetic and environmental factors are likely causes. It can be detected early in pregnancy using ultrasonography. The occurrence of kidney agenesis has been linked to pathogenic variants in three specific genes: ITGA8, GREB1L, and FGF20 (17).

1. **Hypoplasia**

Kidney hypoplasia is a congenital kidney malformation characterized by an abnormally small kidney with a decreased number of nephrons (Figure 2B). Kidney hypoplasia has a significant impact on kidneys function, potentially leading to oligohydramnios syndrome during pregnancy and, ultimately, chronic kidney failure in severe cases (5, 18). This disease can be inherited through dominant and recessive inheritance modes (19). Multiple signaling pathways contribute to kidney hypoplasia. These pathways include the Fibroblast Growth Factor (FGF), Hedgehog (HH), Glial Cell Line-Derived Neurotrophic Factor/Ret (GDNF/Ret), and Paired Box 2 (PAX2) pathways (18).

1. **Dysplasia**

Kidney dysplasia is a kidney malformation that can occur as a result of various dysplastic conditions (Figure 2C), including multicystic dysplastic kidney (MCDK), cystic dysplasia, and obstructive kidney dysplasia (ORD) (18, 20). It can be inherited as an autosomal dominant and is associated with mutations in ITGA8 and FGF20 genes essential for kidney growth and development (21). Furthermore, PAX2 gene mutation or its interaction with p53 pathway can cause kidney dysplasia, leading to impaired nephron induction and abnormal ureteric bud budding from mesonephric duct (20).

Other types of kidney anomalies include ectopic pelvic kidneys, which occur when the kidney fails to ascend to its final position (Figure 2D). Therefore, the kidney will remain in the pelvis, affecting the normal position and function of the kidney (22). A horseshoe kidney occurs when both sides of the vertebral column contain functional kidneys masses fused with uncrossed ureters extending from the kidneys hilum to the urinary bladder (Figure 2E). This malformation affects the kidney’s shape, rotation, position, and vascular supply (23).

### Anomalies of ureter

Later embryonic maldevelopment causes several ureteral defects, including:

1. **Ureteropelvic junction obstruction (UPJO)**

Ureteropelvic junction obstruction is a functional obstruction that causes partial or intermittent complete blockage of urine flow from the kidney, leading to hydronephrosis (Figure 2F) (24). This condition can trigger urinary tract infections or urine retention, resulting in acute kidney injury and even kidneys failure (8). CUPO is a congenital disorder caused by various genes with autosomal dominant inheritance. However, it can also be caused by acquired etiologies resulting in obstruction (25).

1. **Primary nonobstructive non refluxing megaureter kidney**

Primary non-obstructive non-refluxing megaureters, which occur in males and constitute 5 to 10% of all cases of prenatal hydronephrosis, are caused by congenital dilation of the ureter (Figure 2G) (26, 27). Several causes of megaureters include obstruction, reflex, or neither. As part of the diagnostic process, ultrasounds, cystourethrograms, and isotopic renograms apply to patients with this disease (27).

1. **Duplex collecting system**

Duplex kidneys are a rare condition where the kidney pelvis and ureter are duplicated, resulting in a double uretic tip (Figure 2H) (5). This condition is estimated to occur more in females than males. The development of duplex kidneys is caused by a defect in the interactions between the metanephric mesenchymal and the nephric duct, which initiates the ureter. The GDNF/RET signaling axis is a crucial pathway that triggers the RET signaling cascade for the growth of the urinary bladder. The involvement of modifier genes in these cascades could lead to duplex kidney development if disrupted. This condition can lead to several kidneys disorders, including hydronephrosis, pelvic-calyceal dilatation, and cortical scarring (28).

1. **Vesicoureteral reflux (VUR)**

Vesicoureteral reflux (VUR) is a congenital condition where urine flows back from the bladder into the ureters, leading to complications like kidney damage, urinary tract infections, and high blood pressure (Figure 2I) (29). It occurs in 1–2% of the population and can be primary or secondary (30). VUR is caused by ectopic embryonal ureteric budding and is linked to genes like EYA1, ROBO2, RET/GDNF, and PAX2 (31).

### Anomalies of the lower urinary tract

As embryonic development continues, a variety of malformations can occur in the lower urinary tract, including:

1. **Bladder exstrophy**

Bladder exstrophy is a rare congenital malformation that occurs when the bladder develops outside the fetus, causing it to function abnormally (Figure 2J) (32). This condition is caused by a defect in the formation of the cloacal membrane, which eventually ruptures and results in bladder exstrophy (1, 32). Maternal factors like irradiation and smoking in the first trimester can cause severe cases of this malformation. However, folic acid consumption can help reduce the severity of the condition (32).

1. **Bladder agenesis**

Bladder agenesis is a rare condition that results from the failure of the mesonephric duct and ureter to interact during embryogenesis (Figure 2K). It leads to the absence of urine distention and ureteric ectopia, causing complications like kidney dysplasia and agenesis (33, 34). Most cases occur in females, who may maintain kidneys function if their ureters drain into the genital tract. However, male neonates cannot survive unless ureters drain into the rectum or urachus. The prognosis and treatment options for this condition are often poor (34).

1. **Posterior urethral valves**

Posterior urethral valves are abnormal membranes that obstruct urine flow and cause ureter dilation and hydronephrosis (Figure 2L) (8, 35). This condition affects 1 in 5,000 live male births, and 50% of cases progress to chronic kidney failure within 10 years. It can be detected prenatally or after birth, and the severity of the obstruction affects the prognosis (36). Defects in the urinary tract’s development can cause various abnormalities, including duplex kidneys, ectopic tissues, ureteral orifices, and horseshoe kidney (5).

1. **Current diagnostic approaches**

The kidneys system plays a crucial role as an excretory pathway in the human body, and any congenital anomalies in this system can significantly affect an infant’s health. Such anomalies are responsible for approximately 40% of cases of chronic kidney failure in patients who develop the condition within the first three decades of their life. Chronic kidney failure can severely impact the quality of life as it progresses, making early detection and management of such anomalies an essential aspect of patient care (2, 3, 37).

CAKUT encompasses a broad spectrum of kidneys disorders that affect individuals in various ways. While some anomalies can be diagnosed during fetal life through ultrasonography, others may not present until adulthood (3). Genome-wide association studies (GWASs), next-generation DNA sequencing (NGS), and whole exome sequencing (WES) are additional techniques used to diagnose non-syndromic CAKUT. These methods are vital in identifying the underlying genetic causes of the disease (3, 38).

## Conclusion

Congenital Anomalies of the Kidney and Urinary Tract (CAKUT) pose a significant challenge in the healthcare industry, particularly among infants and young children. CAKUT’s complexity arises from multiple environmental, genetic, and epigenetic factors that interfere with normal nephrogenesis. A better understanding of CAKUT’s molecular etiology and genetic causes is essential for identifying the progression causes, improving future prognosis, and providing genetic counseling to patients with CAKUT and their families, which will aid in gene therapy and personalized medicine.

## Author contributions

AM: Conceptualization, Data curation, Formal analysis, Investigation, Methodology, Writing – original draft, Writing – review & editing. IT: Data curation, Writing – review & editing. AT: Writing – original draft, Conceptualization, Data curation, Investigation, Writing – review & editing. RH: Writing – review & editing, Conceptualization, Data curation, Formal analysis, Investigation, Methodology, Project administration, Resources, Supervision, Writing – original draft.
